# Pyomyositis at the surgical site in a patient with chronic myeloid leukemia: a case report and literature review

**DOI:** 10.1186/s12957-016-0873-x

**Published:** 2016-04-19

**Authors:** Katsushi Takebayashi, Hiromichi Sonoda, Tomoharu Shimizu, Hiroyuki Ohta, Hitoshi Minamiguchi, Mitsuaki Ishida, Eiji Mekata, Yoshihiro Endo, Tohru Tani, Masaji Tani

**Affiliations:** Department of Surgery, Shiga University of Medical Science, Seta Tsukinowa-cho, Otsu, Shiga 520-2192 Japan; Department of Hematology, Shiga University of Medical Science, Seta Tsukinowa-cho, Otsu, Shiga 520-2192 Japan; Department of Clinical Laboratory Medicine, Shiga University of Medical Science, Seta Tsukinowa-cho, Otsu, Shiga 520-2192 Japan; Department of Clinical Nursing, Shiga University of Medical Science, Seta Tsukinowa-cho, Otsu, Shiga 520-2192 Japan

**Keywords:** Pyomyositis, Imatinib, Surgical site

## Abstract

**Background:**

Pyomyositis is a rare, subacute, deep pyogenic infection of the muscle tissue. This disease has been previously described in patients that were immunocompromised due to a hematological malignancy.

**Case Presentation:**

A 68-year-old man with a history of chronic myeloid leukemia was treated with imatinib. He was diagnosed with ascending colon cancer and underwent curative surgery. His postoperative course was uneventful, and he was healthy at 6 months after surgery, allowing for reinitiation of imatinib therapy. After the reinitiation of therapy, a computed tomography (CT) scan revealed a mass shadow in the right iliopsoas muscle. This lesion was clinically diagnosed as recurrent colon cancer with an abscess, which was resected surgically. A pathological examination uncovered both edema and inflammation. Two months after the second surgery, imatinib therapy was reinitiated; however, he again developed painful swelling and erythema in his right thigh. A CT scan revealed a similar shadow as described previously. He was then diagnosed with pyomyositis; he underwent incisional drainage and was administered linezolid. Following the treatment for pyomyositis, there was no cancer recurrence or evidence of any recurrent pyomyositis.

**Conclusions:**

Findings from this case suggest that both undergoing surgery and receiving imatinib therapy may modulate an individual’s immune response, whereby the surgical site becomes more prone to infection and may predispose an individual to pyomyositis. The case report is followed by a discussion of the literature regarding this disease, including potential risk factors and the underlying pathogenesis.

## Background

Pyomyositis is a rare, subacute, deep pyogenic infection of the muscle tissue [1]. The disease occurs most commonly in immunocompromised patients, such as those with a hematological malignancy [2]. Without early treatment, pyomyositis can cause significant morbidity and mortality. However, many surgeons are unaware of this disease.

The exact cause of primary pyomyositis remains unclear; however, it is considered the result of hematogenous spread of infection from an occult source. Secondary pyomyositis is usually a consequence of a direct extension from an infectious process. The pathogenesis of the disease is not well understood; however, trauma, malnutrition, viral and parasitic infections, bacteremia, immunodeficiency, and chronic illness are risk factors. Moreover, primary pyomyositis is rare, and the diagnosis of this disease can be delayed if the affected muscle is deeply situated, as local signs may not be apparent in such cases.

In patients with a hematological malignancy, pyomyositis is most commonly seen at the initial presentation of acute lymphoblastic leukemia or during the course of treatment [2, 3]. Cases of pyomyositis have been reported in patients with chronic myeloid leukemia (CML) treated with imatinib [4]. Commonly, immunocompromised patients are at a higher risk of developing pyomyositis. Pyomyositis associated with a surgical site has not been previously reported in postoperative patients.

The present report describes a rare case of pyomyositis at a surgical site in a patient with CML.

## Case presentation

**Fig. 1:**
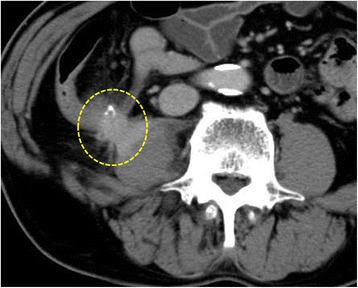
A computed tomography scan reveals a retroperitoneal abscess close to the ileocolostomy. The retroperitoneal abscess and fistula extends from the psoas abscess to the femoral abscess (*encircled by the dotted line*)

**Fig. 2:**
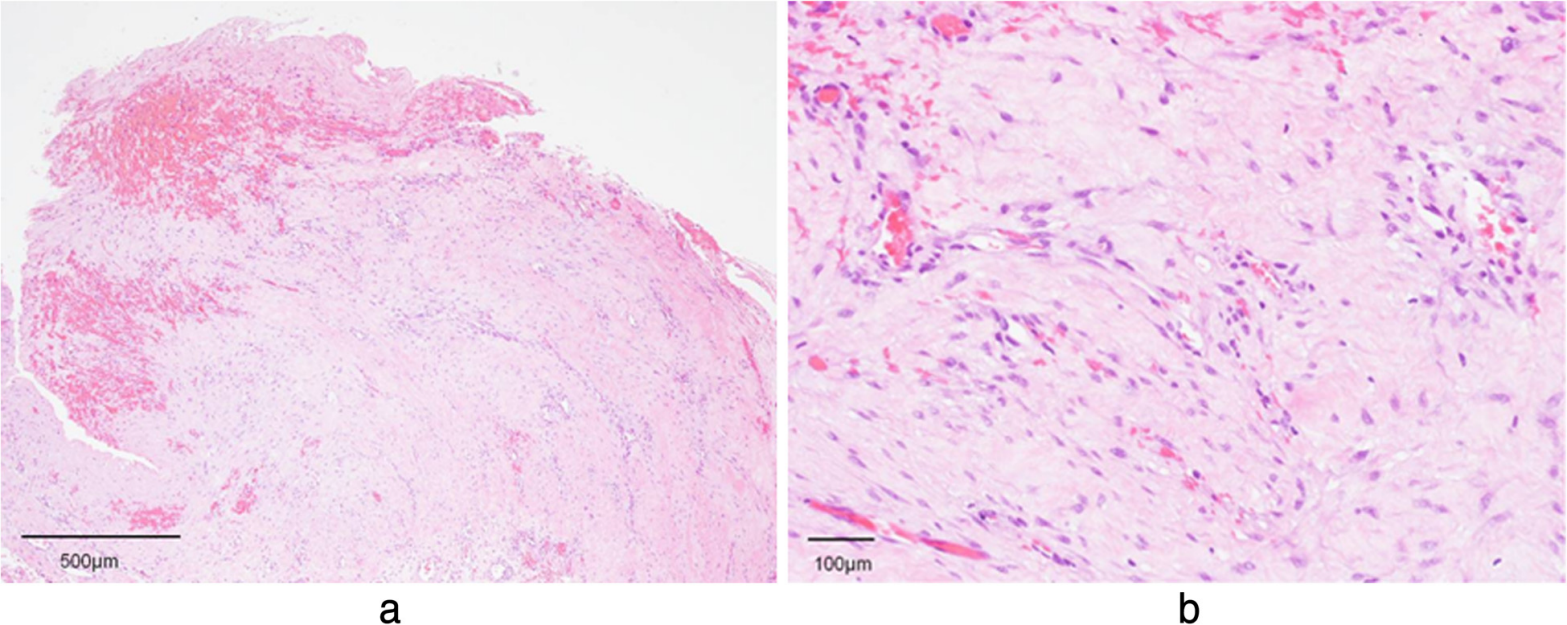
**a** A pathological examination shows granulation tissue with no malignancy. **b** Epidermotropism by lymphocytes and widespread monocellular vacuolization of the epidermis in the granulation tissue (hematoxylin and eosin stain, ×100 magnification)

**Fig. 3:**
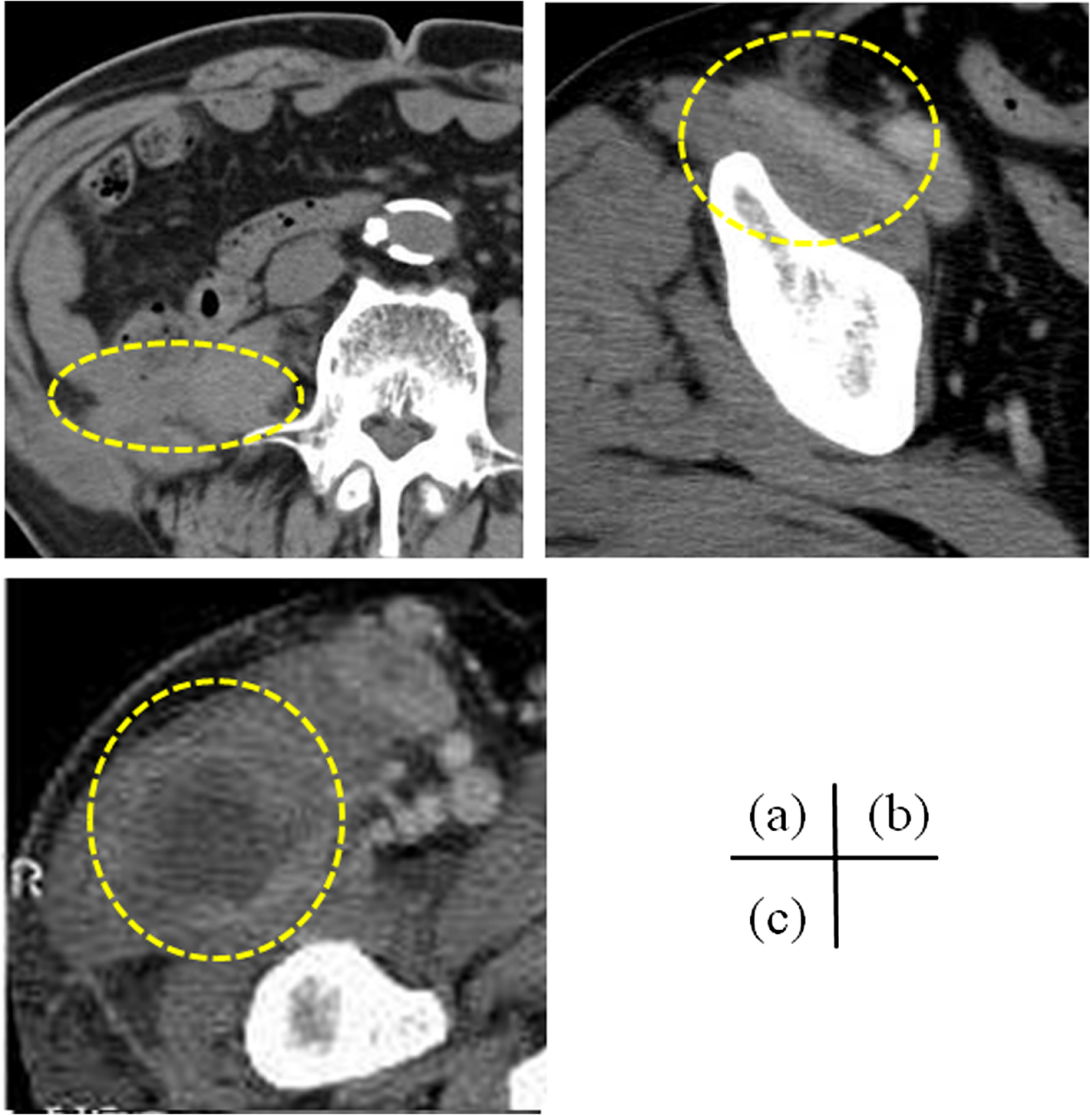
**a**–**c** A computed tomography scan reveals a retroperitoneal abscess 2 months after the surgery for retroperitoneal granulation and abscess. It also shows a fistula extending from the psoas abscess to the femoral abscess, as in Fig. 1 (*encircled by the dotted line*)

A 68-year-old man was diagnosed with CML in 2009. His complete blood cell count at the initial diagnosis was as follows: a white blood cell (WBC) count of 24,500 cells/μL, neutrophil bands of 64 %, hemoglobin level of 10 g/dL, platelet count of 537,000 cells/μL, and a lactate dehydrogenase (LDH) level of 346 IU/L. He has been treated with 300 mg of imatinib once a day. After 6 months, a complete cytogenetic response was achieved and the 300-mg imatinib therapy with was continued. In April 2010, he was diagnosed with T2N0M0, stage I ascending colon cancer, and underwent curative surgery with regional lymph node dissection without adjuvant chemotherapy. His postoperative course was uneventful, and he was healthy at 6 months after surgery, allowing for reinitiation of the imatinib therapy (300 mg/day). In November 2010, a computed tomography (CT) scan revealed a mass shadow in the right iliopsoas muscle that was contiguous with the site of the ileocolostomy (Fig. 1). His body temperature was 37.2 °C, and the results of his routine laboratory examination were as follows: WBC count of 9700 cells/μL, C-reactive protein (CRP) level of 16.7 mg/dL, and LDH and creatine kinase (CK) levels within the normal range. A CT-guided biopsy and total colonoscopy did not demonstrate the presence of malignant cells, and no bacteria were isolated from the drained fluid culture. After 6 months, a CT scan revealed that the mass shadow had enlarged. This lesion was clinically diagnosed as recurrent colon cancer with an abscess, which was then resected surgically. A pathological examination uncovered both edema and inflammation of the fascia and muscular tissues with the presence of lymphocytes, neutrophils, plasma cells, and foreign-body giant cells. The pathological diagnosis was granulation tissue with no malignancy (Fig. 2a, b). The patient’s postoperative course was good, and he was healthy without recurrence at 2 months after the second surgery, allowing for reinitiation of imatinib therapy (300 mg/day). However, he developed painful swelling and erythema on the right thigh after reinitiating therapy. His body temperature was 37.4 °C, and the results of his routine laboratory examination were as follows: WBC count of 7100 cells/μL, CRP level of 1.93 mg/dL, and LDH and CK levels within normal range. A CT scan revealed a mass shadow extending from the right iliopsoas muscle to the right thigh muscle 2 months after the surgery for retroperitoneal granulation and abscess (Fig. 3a–c). A femoral muscle biopsy showed no malignancy. No bacteria were isolated from the blood and drained fluid cultures. Despite the administration of several antibiotics, the patient improved after starting an oral linezolid therapy and undergoing incisional femoral muscle drainage. The routine laboratory examination results were within normal range with values as follows: a WBC count of 4500 cells/μL, CRP level of 0.2 mg/dL, and LDH and CK levels within normal range. After that, he no longer received oral imatinib therapy. There was no cancer recurrence or evidence of any recurrent pyomyositis 2 years after his recovery from final pyomyositis occurrence.

### Discussion

This report described a case of pyomyositis at the surgical site that occurred in the context of a recurrent iliopsoas abscess in a patient with CML. Pyomyositis is a rare and rapidly progressive complication of hematological disorders and can be a focus of infection [3]. Hayashi et al. described three cases of pyomyositis in patients with hematological disorders, and they were all associated with methicillin-resistant *Staphylococcus aureus* (MRSA). They also described *S. aureus* as the most common pathogen associated with pyomyositis. However, its identification from blood or fluid culture can be difficult. MRSA pathogens were isolated from blood culture in 5 to 38 % of cases [3]. In our case, we performed several blood and drained fluid cultures. However, no bacteria were isolated from either the drained fluid or blood cultures at any time. From this point, MRSA was considered to be the pathogen in our case, so we administered linezolid. In the early stage of pyomyositis, treatment with antibiotics alone can be effective for local infection control. However, if abscess formation has begun, the combination of surgical drainage and medical intervention is the chosen treatment regimen and usually leads to a complete recovery.

There might be other factors related to pyomyositis aside from bacterial infection. Some cases of pyomyositis and fasciitis without bacterial infection have been described [4, 5]. However, a case of pyomyositis associated with a surgical site has not been previously described (Table 1). In the present case, CML was initially treated with imatinib therapy, and the patient experienced infectious episodes after the reinitiation of imatinib therapy in both instances. Imatinib is a selective inhibitor of ABL, c-kit, and platelet-derived growth factor (PDGF) kinases. Imatinib can modulate immunological homoeostasis in vivo, explaining its wide and increasing range of known immune side effects [4]. Common adverse effects of imatinib include edema, nausea, vomiting, diarrhea, muscle cramps, and cutaneous reactions, which are accompanied by emergence of a circulating T cell clone and autoantibodies against the nucleus. Jardin et al. suggested that imatinib should be included in the group of pseudolymphoma-inducing drugs [5]. Edema and fluid retention are characteristic side effects of imatinib that possibly result from the alteration of interstitial fluid pressure control via the PDGF receptor [6]. We revealed that surgery induces the production of adhesion molecules, cytokines, vascular endothelial growth factors, immunosuppressive agents, and TGF-β [7]. From these points of view, we hypothesize that inflammation was first observed at the surgical site, and surgery-induced factors, including inflammatory mediators or angiogenic factors, became concentrated at the surgical site. In the context of these surgical factors, pyomyositis might have been induced by imatinib.

**Table 1:** Reported cases of pyomyositis in patients with chronic myeloid leukemia treated with imatinib

Case no.	Authors	Year	Age (years)	Gender	Site of muscle involvement	Pathogen causing pyomyositis	Medical treatment	Imatinib treatment during pyomyositis	Surgical treatment	Patient outcome
1	Jardin et al. [5]	2005	70	Male	Left thigh	None	Oxacillin	Switch to hydroxyurea	Incision and drainage	Good
2	Chen et al. [4]	2011	17	Male	Bilateral thigh	None	No antibiotics	Discontinuation	None	Good
3	Our case	2016	68	Male	Right thigh	None	Linezolid	Discontinuation	Incision and drainage	Good

## Conclusions

Our findings from this case suggest that both undergoing surgery and receiving imatinib therapy may modulate an individual’s immune response, whereby the surgical site becomes more prone to infection and may predispose an individual to pyomyositis. It is necessary for surgeons to recognize pyomyositis as a possible postoperative complication at the surgical site in immunocompromised patients.

### Consent

Written informed consent for publication of this case report and any accompanying images was obtained from the patient. A copy of the written consent is available for review by the Editor-in-Chief of this journal.

## Abbreviations

CML: chronic myeloid leukemia
CT: computed tomography
MRSA: methicillin-resistant *Staphylococcus aureus*
PDGF: platelet-derived growth factor
